# Brain connectivity networks underlying resting heart rate variability in acute ischemic stroke

**DOI:** 10.1016/j.nicl.2023.103558

**Published:** 2023-12-19

**Authors:** Violeta Dimova, Claudia Welte-Jzyk, Andrea Kronfeld, Oliver Korczynski, Bernhard Baier, Nabin Koirala, Livia Steenken, Bianca Kollmann, Oliver Tüscher, Marc A. Brockmann, Frank Birklein, Muthuraman Muthuraman

**Affiliations:** a Department of Neurology, University Medical Center of the Johannes Gutenberg University Mainz, Mainz, Germany; b Department of Neuroradiology, University Medical Center of the Johannes Gutenberg University Mainz, Mainz, Germany; c Edith-Stein Fachklinik for Neurorehabilitation, Bad Bergzabern, Germany; d Haskins Laboratories, Yale University, New Haven, CT 06511, USA; e Leibniz Institute for Resilience Research (LIR) gGmbH, Mainz, Germany; f Department of Psychiatry and Psychotherapy, University Medical Center of the Johannes Gutenberg University Mainz, Mainz, Germany; g Institute for Molecular Biology (IMB), Mainz, Germany; h Neural Engineering with Signal Analytics and Artificial Intelligence, Department of Neurology, University of Würzburg, Würzburg, Germany

**Keywords:** Stroke, Heart rate variability, Low-frequency spectral band, Voxel-based lesion mapping, Connectivity network analysis

## Abstract

Acute strokes can affect heart rate variability (HRV), the mechanisms how are not well understood. We included 42 acute stroke patients (2–7 days after ischemic stroke, mean age 66 years, 16 women). For analysis of HRV, 20 matched controls (mean age 60.7, 10 women) were recruited. HRV was assessed at rest, in a supine position and individual breathing rhythmus for 5 min. The coefficient of variation (VC), the root mean square of successive differences (RMSSD), the powers of low (LF, 0.04–0.14 Hz) and high (HF, 0.15–0.50 Hz) frequency bands were extracted. HRV parameters were z-transformed related to age- and sex-matched normal subjects. Z-values < -1 indicate reduced HRV. Acute stroke lesions were marked on diffusion-weighted images employing MRIcroN and co-registered to a T1-weighted structural volume-dataset. Using independent component analysis (ICA), stroke lesions were related to HRV. Subsequently, we used the ICA-derived lesion pattern as a seed and estimated the connectivity between these brain regions and seven common functional networks, which were obtained from 50 age-matched healthy subjects (mean age 68.9, 27 women). Especially, LF and VC were frequently reduced in patients. ICA revealed one covarying lesion pattern for LF and one similar for VC, predominantly affecting the right hemisphere. Activity in brain areas corresponding to these lesions mainly impact on limbic (r = 0.55 ± 0.08) and salience ventral attention networks (0.61 ± 0.10) in the group with reduced LF power (z-score < -1), but on control and default mode networks in the group with physiological LF power (z-score > -1). No different connectivity could be found for the respective VC groups. Our results suggest that HRV alteration after acute stroke might be due to affecting resting-state brain networks.

## Highlights

• Acute cerebral stroke leads to abnormal reductions in low frequency bands of HRV.
• Associated lesions location in the right temporo-parieto-occipital cortex, basal ganglia, thalamus, and interconnections.
• Acute brain lesions related to abnormal HRV impact resting state cortical networks.
• Abnormal low frequency bands associate with the limbic and the salient ventral attention network.

## 1 Introduction

Autonomic nervous system function is regulated by widely ramified networks of the central nervous system involving the brain and the spinal cord. Key areas encompass preganglionic sympathetic neurons from T1 to L2 thoracolumbar segments, the nucleus of the solitary tract, the rostral ventrolateral medulla as well as anterior limbic circuits, the latter also as a part of emotional and stress-related autonomic regulation (Gibbons, 2019). Brain areas involved in autonomic cardiovascular control include thalami, insulae, amygdalae, cingulate cortex, hippocampus, the right angular and bilateral supramarginal gyri, the hypothalamus, midbrain and brainstem.

It has been shown several times that very differently located focal brain lesions such as strokes can lead to disturbances in cardiac autonomic function, i.e. altered sympathetic and parasympathetic responses (Akil et al., 2015, Al-Qudah et al., Oct 2015, Jimenez-Ruiz et al., May 2021, Mo et al., Feb 2019, Xiong et al., Jan 2018), sometimes leading to arrhythmia or cardiac failure. Such disturbances can be quantified by assessing the heart rate variability (HRV) (Akil et al., 2015, Ha et al., Jul 2018, Hilz and Dutsch, Jan 2006), which was reported to be reduced by acute strokes (Korpelainen et al., Nov, 1996). HRV after stroke can be determined during normal breathing by time-domain parameters such as the coefficient of variation (VC) and the root mean square difference of successive differences (RMSSD) of R-R intervals, or by parameters in the frequency-domain such as the spectral power of low (LF, 0.04–0.14 Hz) and high (HF, 0.15–0.50 Hz) frequency bands of trigonometric regressive spectral analyses. Theoretically, parameters in the frequency domain permit conclusions about the influences of the sympathetic and parasympathetic nervous systems on HRV. Changes in the HF band are thought to represent mainly parasympathetic influences and correlate with the parameters in the time domain, whereas the LF band represents both sympathetic and parasympathetic influences on HRV (McCraty and Shaffer,Jan, 2015).

There seems to be a right hemispheric dominance in HRV control. Recent lesion studies describe HRV alteration in the time domain more often in relation to damage to right versus left cortical and subcortical brain regions despite similar clinical outcomes (Raphaely-Beer et al., 2020). This recent study also demonstrated that different parameters of HRV measurement were associated with different brain damage in voxel-based lesion symptom mappings. However, the mechanisms how the very different focal brain lesions can induce changes of HRV are still unknown.

Fundamental work has shown that the control which the brain exerts on heart rate variability in healthy subjects can only be understood if network-based statistics were applied simultaneously to datasets of gray-matter morphometric, white-matter tractographic and functional connectivity networks (Ruffle et al., Oct, 2021). We therefore hypothesize that not the single brain lesion determines HRV after stroke but the effect of that brain lesion on brain networks. In the present study we chose an approach which first determines which brain lesions have an influence on HRV in parallel independent component analyses (ICA). In a second step we sought to investigate whether these ICA derived lesions could be connected to common functional brain networks, which were assessed in age-matched healthy subjects (Damoiseaux, 2017). We have chosen to use an approach with seven functional networks (Schaefer et al., 2018). If our hypothesis is true, we expect, that the ICA lesion pattern, which is correlated to HRV, affects these brain networks differently than lesion patterns without affecting HRV.

## 2 Materials and methods

### 2.1 Subjects and study design

#### 2.1.1 Stroke patients

Patients with acute first-ever ischemic stroke at any cortical localization (n = 42; 16 women; mean age 65.7 ± 13.1 y; 2–7 days post-stroke) were recruited from the Department of Neurology, University Medical Center Mainz (Mainz, Germany) as part of a single-center project funded by the German Research Foundation (Deutsche Forschungsgemeinschaft (DFG)). Exclusion criteria for all patients were previous strokes or other lesions of the central nervous system, missing ability to provide informed consent or pre-existing cardiac arrhythmia precluding HRV analysis.

Patients underwent standardized neurological examination and assessment of HRV. Patients' post-stroke National Institute of Health Stroke Scale score (NIHSS, max. 42 points) and the actual antihypertensive medication were recorded.

#### 2.1.2 Healthy controls for behavioral data

Healthy controls (n = 20; 10 women; mean age 60.7 ± 10.3 y) were enrolled by meeting the following criteria: i) age > 50 years; ii) no chronic medical condition; iii) no intake of drugs for at least one week prior to examination except contraceptives, vitamins, and thyroid hormone-substitution; iv) no current or past neurologic or psychiatric disease. Healthy controls had no antihypertensive medication and no diseases known to influence HRV.

#### 2.1.3 Standard protocol approvals and patient consents

The study followed the Declaration of Helsinki and was approved by the Ethics Committees of the Rhineland Palatinate Medical Association (No. 837.032.17 (10866)), Germany. Informed written consent was obtained from patients and healthy controls.

### 2.2 Data acquisition

#### 2.2.1 Heart rate variability (HRV)

HRV at rest was recorded during 5 min with standard electrocardiogram (ECG) electrodes (FAN, Schwarzer, Germany) attached to the extremities (Haegele-Link et al., 2008). The patients sat in a reclining position with their limbs resting at heart level and breathed in their individual rhythmus. Ectopic heat beats were cleaned-up automatically by the software and manually after inspection, if necessary.

As time domain parameters, the VC and RMSSD were calculated. As frequency domain parameters, the low (LF, 0.04–0.14 Hz) and high (HF, 0.15–0.50 Hz) frequency bands of R-R intervals (RRI) were standardly analyzed by Fast Fourier transform-based approach (FFT). The magnitude of LF and HF oscillations was determined as the integral under the power spectral density curves of RRI (ms^2^/Hz) for the LF and HF frequency bands, and was expressed as LF and HF powers of RRI (ms^2^) (Hilz and Dutsch,Jan, 2006).

#### 2.2.2 Structural MRI of patients

MRI scans were acquired 2–7 days after stroke onset as part of a clinical routine examination. Scanning was performed either on a 3 Tesla Magnetom Skyra or a 1.5 Tesla Magnetom Sola (Siemens Healthineers, Erlangen, Germany) with a phased array head/neck coil. High-resolution T1-weighted anatomical scans (repetition time (TR) = 2.25 s; echo time (TE) = 3.83 ms; flip angle = 9°; 144 slices per slab; 1 mm^3^ isotropic voxel size) were obtained to improve the spatial normalization of the lesion-positions onto the Montreal Neurological Institute (MNI) brain template. The stroke lesions were marked on diffusion-weighted sequences of routine structural MRI scans using MRIcroN (Rorden et al., Jul, 2007) and exported in an SPM-readable format. The lesion volumes were co-registered together with the diffusion-weighted images to the volume-dataset using SPM 12 (The Welcome Centre for Human Neuroimaging, UCL Queen Square Institute of Neurology, London, UK), which is designed to work with MATLAB (The MathWorks, Inc.). Both the MRI scan and the lesion shape were mapped into stereotaxic space using the normalization algorithm of SPM12. This normalization effectively aligns the shape and size of each individual’s brain lesion to the same stereotaxic spacing (Baier and zu Eulenburg, 2014).

#### 2.2.3 Resting-state functional MRI (rs-fMRI) of age-matched healthy controls

RS-fMRI data of age stratified healthy controls (n = 50; 27 women; mean age 68.9 ± 4.7 y) from the AgeGain Study Group were used for functional connectivity analyses. Subjects were scanned using a 3 T-MRI scanner, TrioTim Magneton (Siemens Medical Systems, Erlangen, Germany). Anatomical scans were captured using a T1-weighted Magnetization Prepared Rapid Gradient Echo (MPRAGE) sequence with the following parameters: sagittal slices = 176, scan time = 4.18 min, repetition time (TR) = 1900 ms, echo time (TE) = 2.52 ms, flip angle = 9°, field of view (FOV) = 250 mm, and voxel volumes = 1.0 × 1.0 × 1.0 mm. During the resting-state functional MRI (rs-fMRI) examination, participants were instructed to keep their eyes closed without thinking of anything in particular or falling asleep. T2-weighted scans were captured with the following parameters: Echo planar imaging (EPI) multiband sequence scan time = 11.02 min, transversal slices = 60, slice thickness = 2.5 mm, TR = 1000 ms, TE = 29.0 ms, flip angle = 56°, and FOV = 210 mm, multiband acceleration factor = 4.

The 50 rs-fMRIs were pre-processed by the program Data Processing Assistant for Resting-State fMRI (DPARSFA (Chao-Gan and Yu-Feng, 2010) implemented in MATLAB (MATLAB, 2016). After the removal of the first six images, we applied a series of steps including slice timing correction and realignment to eliminate the influence of head motion. All scans were checked for excessive head motion; participants did not show head motion more than 3 mm. The realigned images were segmented into gray matter (GM), white matter (WM), and cerebrospinal fluid (CSF), spatially normalized to MNI space using Diffeomorphic Anatomical Registration Through Exponentiated Lie Algebra (DARTEL (Ashburner, 2007), and resampled to 3 × 3 × 3 mm voxels. To reduce the influence of noise, we regressed out linear trend, 12 motion parameters, WM, CSF, and global signal as nuisance regressors. Later, the functional images were filtered with a bandpass filter between 0.1 and 0.01 Hz and smoothed with a 6-mm Gaussian kernel.

### 2.3 Data analysis

#### 2.3.1 Behavioral data

Behavioral data were analyzed using the SPSS software package (version 19 for Windows; IBM SPSS Inc., Chicago, USA). Statistical significance was defined at an alpha level of < 0.05.

Group differences (patients vs. controls) were tested for age and HRV parameters by single one-way analysis of variance (ANOVA); for distribution of sex by cross-tabulation statistics. Right versus left hemisphere stroke patients, independent of lesion location, were compared regarding HRV parameters using one-way ANOVA.

Some patients were regularly medicated by beta blocker (n = 2) or a combination of beta blocker and other antihypertensive drugs (n = 11), or by antihypertensive drugs excluding beta blocker (n = 19). The remaining 10 patients were not treated with antihypertensive drugs. Differences in each HRV parameter between the patient groups with and without antihypertensive medication, and healthy controls were analyzed by one-way ANOVAs (post-hoc group comparisons based on LSD estimation).

The measures of heart rate variability are strictly age dependent. For creating a homogeneous data space, scores of VC, RMSSD, LF and HF bands were z-transformed based on the mean and standard deviation of the scores of the age-matched healthy subjects. Z-values of < -1, indicating 1 standard deviation lower than the mean of healthy controls, were considered “abnormal”. We only considered reduction of HRV of abnormal because there is no upper limit for HRV. The quantification of z-scores showed the highest frequency of impairment in the VC and in the LF bands. Therefore, subsequent lesion pattern and network analyses contrast patients having reduction of VC or LF band versus all other patients.

#### 2.3.2 Identification of lesion patterns by parallel independent component analysis (ICA)

Independent component analysis (ICA) allows data-driven identification of hidden non-correlating components that underlie sets of measurements. The parallel ICA algorithm implemented in the fusion ICA toolbox (Calhoun et al., Jan, 2006) was used to discover independent components (lesion patterns) from two inputs, here, namely the VC and LF values for each patient is the first input, and the lesion volumes (with actual spatial patterns without binarizing) derived from the MRI of the same patient is the second input. Two separate parallel ICAs were performed for the z-transformed continuous measures of VC and LF band as these parameters showed abnormal changes compared to the healthy controls.

ICA optimization was based on the Infomax algorithm (Anemüller et al., Nov, 2003), which maximizes the mutual entropy to enhance the independence between the components for the two inputs. Finally, to avoid overfitting because of too many estimated parameters, the learning rate of the correlation term is adaptively adjusted (Liu et al., Jan, 2009). To determine the correct number of independent components, a modified version of the Akaike information criterion proposed by Li et al. (Li et al., Nov, 2007) was applied. The components from each modality were selected to be the correlations with the highest significance. First, we used the Akaike information criterion to estimate the number of components and then to reduce the component number to reach a consistent level among the different test runs.

The most frequently lesioned brain areas from the obtained lesion pattern were estimated using FSL (version 6.0.1) (Woolrich et al., Mar 2009, Smith et al., 2004, Jenkinson et al., 2012). First, lesion volume maps from each patient thresholded at t-value > 3 were combined (using *fslmaths command*) to form a single lesion map for the whole group. This obtained image was then masked (using *fslmaths command*) by overlaying three different atlases (John Hopkins University white matter atlas (Wakana et al., 2007, Hua et al., 2008, Mori et al., 2015), Harvard Oxford cortical and subcortical atlas (Makris et al., Apr 2006, Frazier et al., Jul 2005, Desikan et al., 2006, Goldstein et al., 2007) to obtain the volume (using *fslstats command*) at each region of interest (ROI). Lesion areas with cluster sizes greater than 10 voxels were estimated. The percentage overlay was obtained by taking the ratio of the lesion volume at a ROI to the total lesion volume.

#### 2.3.3 Directed connectivity analysis using time resolved partial directed coherence

For directed connectivity analysis, the lesion pattern for VC and LF, which were identified by the ICA, were seeded into the rs-fMRIs of healthy subjects (cohort of the German Age Gain Study Group described above). Connectivity analyses were performed for the patient groups with and without reduced HRV separately. The Schaefer connectivity atlas (Schaefer et al., 2017), which parcellates the brain into seven networks (visual, somatomotor, dorsal attention, salience ventral attention, control, limbic and default network), was used for defining the target networks for connectivity estimation. The time series was extracted from the active voxels of the lesion pattern to form a pooled time series and estimate the connectivity between this seed region and the seven networks using time resolved partial directed coherence (TPDC).

TPDC allows focusing on the temporal dynamics of the signal and analyze the directed connectivity at a specific frequency (Anwar et al., 2013). It adopts the dual­extended Kalman filter (DEKF) (Wan and Nelson, 2001) to estimate time­dependent autoregressive coefficients. In brief, DEKF in the TPDC is a predictor-­corrector algorithm that estimates the state of a process, i.e. functional connection. At each time point, one extended Kalman filter estimates the state and shares it with the other, the second one estimates the model parameters and feeds back to the first. By using two extended Kalman filters in parallel, we can estimate both state and model parameters of the system at each observed data point in the time series. Subsequently, using the Fourier transform of the estimated time­dependent multivariate autoregressive (MVAR) coefficients, partial directed coherence (PDC) can be calculated, as described in (Baccalá and Sameshima,Jun, 2001). Prior to fitting any model to observed data, it is necessary to estimate the optimum number of model parameters. Choosing too few parameters may miss the true system dynamics, however, applying a higher model order than necessary may cause spurious results. Hence the best trade-off between model accuracy and number of parameters must be met. The model order to estimate the MVAR coefficients was fixed for all subjects to be 5. In order not to overestimate we checked the model order with the Akaike information criterion (AIC). Akaike improved his definition of FPE by introducing the minimization of the Kullback-Leibler information entropy, which is the distance between the fitted model and true data set (Liddle, 2007). The best model is the one which gives minimum AIC value (Akaike, 1974). After squaring the partial directed coherence (PDC) value, the normalized value falls between 0 and 1. By calculating PDC with estimated MVAR coefficients at each time point, the connectivity matrices corresponding to the time series were obtained. In the rs-fMRI dataset of the healthy subjects, we extracted frequency band of interest from 0.009 to 0.08 Hz and averaged the outcome across each time point to obtain robust connectivity values between brain regions. (Anwar et al., 2016) The choice of this frequency range is based on several factors. Firstly, it is known that neuronal activity in the brain exhibits low-frequency oscillations (<0.1 Hz) and believed to reflect functional networks that are active in that region during task performance (Greicius et al., 2004, Biswal et al., Sep 2010). Secondly, the BOLD signal is relatively slow, with changes occurring over several seconds, which is believed to be related to underlying neuronal activity and functional connectivity in the brain (Fox and Raichle,Sep, 2007). The low-frequency range (0.009–0.08 Hz) has been used in numerous fMRI studies to investigate functional connectivity in the human brain (Fox and Raichle, Sep 2007, Ding et al., 2022). After estimating the TPDC values, the significance level was calculated from the applied data using a bootstrapping method (Kamiński et al., Aug, 2001). In short, we divided the original time series into smaller non-overlapping windows and randomly shuffled the order of these windows to create a new time series. The TPDC value is calculated based on a randomly shuffled time series for 1,000 times and the 99th percentile of the connectivity value was taken as the significance threshold. This process is performed separately for each subject. The resulting value was the significance threshold value for all connections. The open source MATLAB package autoregressive fit (ARFIT) (Neumaier and Schneider, 2001, Schneider and Neumaier, 2001) was used for estimating the autoregressive coefficients from the filtered signals. We applied the time reversal technique (Kamiński et al., Aug 2001, Haufe et al., 2013) as a second significance test on the connections already identified by TPDC using data-driven bootstrapping surrogate significance test. In addition, we have added the surrogate data based on the method for amplitude adjusted Fourier transform (AAFT) algorithm to generate the surrogate data as shown in the supplementary Fig. 1 (in black) (Theiler et al., 1992). We estimated 1000 realizations of this new method and estimated the significance threshold with the 99th percentile which was the values with the range of TPDC (0.08–0.095) indicating the threshold initially used was 0.1 appropriate for defining the significant connections in this study. Medication status of patients (i.e. “hypertensive medication with beta blocker”, “other hypertensive medication” and “no medication”) was included as covariate in the connectivity analyses.

**Fig. 1:**
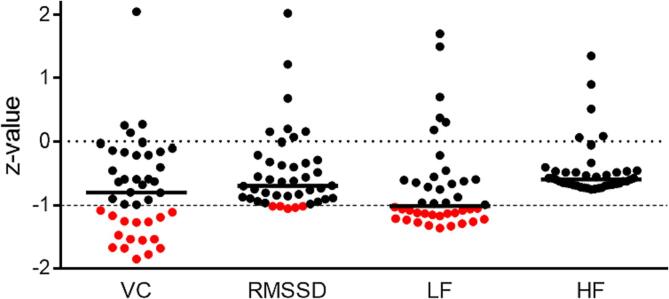
**Distribution of z-transformed scores of the HRV parameters**. LF z-values < -1 represent patients with abnormal, low sympathetic tone, whereas a z-value > -1 those within the norm. Each dot represents one patient, red indicates abnormal values. (For interpretation of the references to colour in this figure legend, the reader is referred to the web version of this article.)

#### 2.3.4 Validation of connectivity using support vector regressor (SVR) prediction analyses

For validation of the effectiveness of the connectivity values, we further applied support vector machine (SVM) analysis to predict the LF and VC scores used in the criteria for the patient group with abnormal changes and the group without. We performed a support vector regressor (SVR) analysis—a machine-learning-based multiple-regression method—that could associate the observed and trained values and determine the prediction accuracy (Drucker et al., 1996). Here, we used the polynomial function kernel for this projection due to its good performance as discussed in (Cortes and Vapnik) and use the grid search (min = 1; max = 10) to find the few optimal input parameters and gamma (0.25). The selection was checked by taking 75 % of the data for training and 25 % for testing. To obtain the threshold for prediction accuracy, we devised an approach based on the statistical inference obtained from the Bayesian credible interval (Curran, 2005). The 75 % threshold could differentiate the posterior distribution from the 95 % Bayesian credible interval (indicating the inclusion of 95 % of the data points). Here, the posterior distribution and the credible interval were obtained considering all connectivity values from all subjects from the group with abnormal values and the remaining patients and the highest density interval containing 95 % (range: 0.15–0.64) of the distribution. Hence, the prediction accuracy of the above (75 %) obtained after 10-fold cross-validation was considered a significant result.

## 3 Results

### 3.1 Clinical characteristics of stroke patients

Patient demographic characteristics, stroke location and size, and medical history are displayed in Table 1. Mean lesion volume was 11.23 ml, range 0.14–97.5 ml. The National Institutes of Health Stroke Scale (NIHSS) mean score after acute medical/surgical intervention was 3.0 (range 0–10 points). Patients had hypertension in 74 %, hyperlipidemia in 29 %, diabetes mellitus in 31 % and were obese in 26 %; 36 % had a history of nicotine abuse relevant for the stroke. A clear separation of the sample could be made regarding antihypertensive drugs: n = 13 patients had a combination therapy including beta blockers, n = 19 without beta blockers; n = 10 patients did not take antihypertensive medication at the time of investigation. For all HRV parameters, no difference in scores was found between right (n = 26) and left hemispheric strokes (n = 16) (ANOVA: F(1,0) = [0.0–0.68], p > 0.41). No differences were found also for age (ANOVA: F (1,0) = 2.39, p = 0.13) and the distribution of sexes (χ = 0.79, p = 0.38) between the patients and healthy controls.

**Table 1:** Demographic, imaging and clinical characteristics of the study cohort.

Demographic		N = 42
Age, years, mean ± SD		65.7 ± 13.1
Female, n (%)		16 (38.1)
Imaging		
Stroke location, n (%)	ACA	1 (2.4)
	MCA	26 (61.9)
	PCA	9 (21.4)
	ACA + MCA	3 (7.1)
	MCA + PCA	3 (7.1)
Right hemispheric stroke, n (%)		26 (61.9)
Lesion volume, mean ± SD, range	ml	11.23 ± 18.94 (0.14–97.50)
Clinical		
NIHSS after intervention, median, range		3.0, 0–10
Paresis after intervention, n (%) Muscle strength grading (MRC)	Severe/total dysfunctionMRC score 5 (complete paralysis)	5 (11.9)
	Slight/moderate weaknessMRC score1-4	15 (35.7)
	no dysfunction MRC score 5	22 (52.4)
Vascular risk factors		
Hypertension, n (%)		31 (73.8)
Hyperlipidemia, n (%)		12 (28.6)
Diabetes mellitus, n (%)		13 (31.0)
Adipositas, n (%)		11 (26.2)
Nicotine abuse, n (%)		15 (35.7)

ACA, A. cerebri anterio; MCA, A. cerebri media; PCA, A. cerebri posterior; NIHSS, National Institute of Health Stroke Scale score.

### 3.2 Differences in resting HRV parameters in stroke patients versus healthy controls

HRV of patients was lower than of healthy controls (ANOVA: F (1,0) = [5.20–10.64, p < 0.026). (Supplementary Fig. 2). Patients were classified regarding hypertensive medication into the groups “beta blocker”, “other hypertensive medication” and “no medication” because of the potential effect of this medication on HRV. The patient groups were contrasted to the healthy controls. There was a main effect of group on VC (ANOVA: F(3,0) = 3.40, p = 0.024) and LF (F(3,0) = 3,87, p = 0.014), but not on RMSSD (F(3,0) = 2.58, p = 0.062) or HF (F(3,0) = 2.43, p = 0.074). Post-hoc comparisons are displayed for VC und LF in Supplementary table 1; all parameters including VC and LF did not differ between the medication-related patient groups. On a descriptive level, the patient group without medication displayed the lowest scores in VC and LF, further making a significant effect of beta blocker medication on HRV unlikely in the present investigation.

**Fig. 2:**
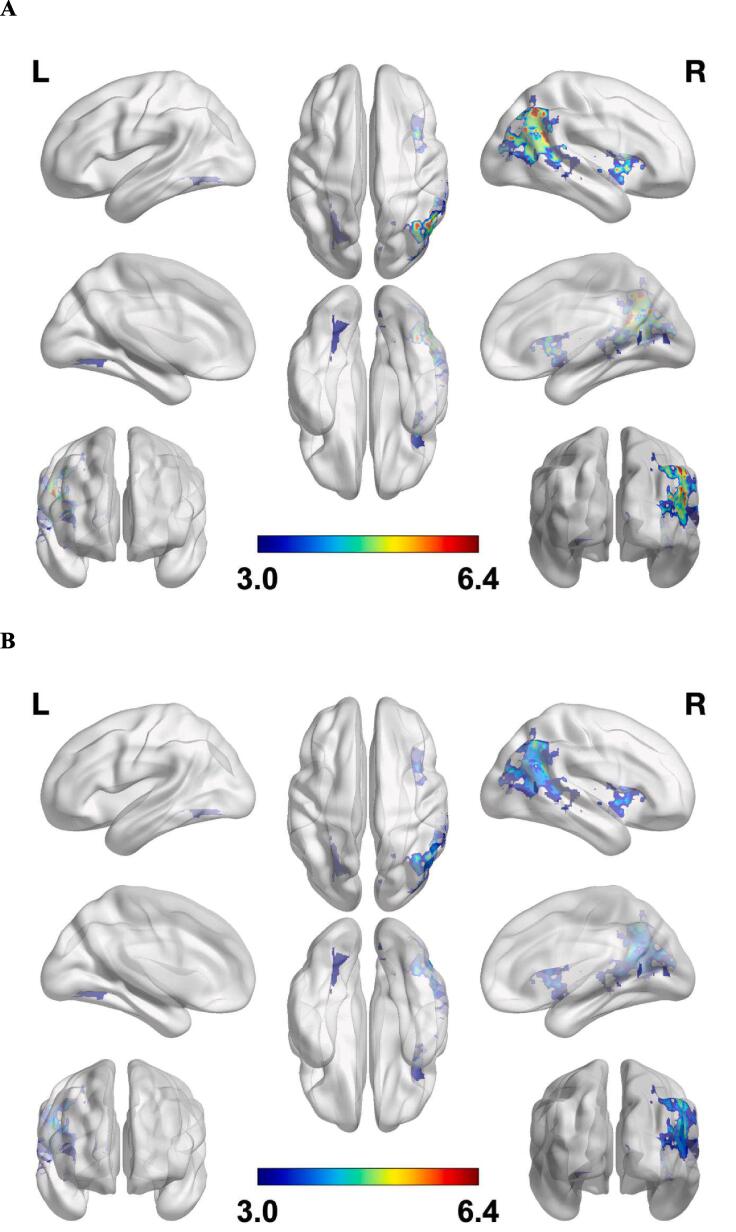
**Cortical lesion pattern from ICA analyses.** The strength of the pattern is depicted by the colour bar, with warmer red colours indicating highest t-value. **A.** Cortical lesion pattern derived from the independent parallel ICA analyses for the low frequency band LF as covariate. **B.** Cortical lesion pattern derived from the independent parallel ICA analyses for the variation coefficient VC as covariate. (For interpretation of the references to colour in this figure legend, the reader is referred to the web version of this article.)

### 3.3 Abnormal changes in resting HRV in stroke patients

Fig. 1 shows the results of the z-values of the HRV parameters. The highest frequency of patients with abnormal impairment (z-score < -1) was found for LF (n = 21) and VC (n = 16). From the patients with abnormal LF, 13 patients displayed also abnormal scores in VC. For imaging analyses, the LF and VC groups with values < -1 were contrasted to the patients with values > -1.

### 3.4 Brain lesion pattern associated with LF and VC

The lesion distribution and the z-scores of LF and VC from all patients were included as two inputs into two separate parallel ICAs. The ICAs revealed for both VC and LF two very spatially similar and significantly covarying components (VC: r = 0.64, p < 0.001; LF: r = 0.61, p < 0.001). The brain lesion pattern comprised predominantly lesions in the right hemisphere (Fig. 2A-B). Table 2 shows the cortical, subcortical and white matter projections lesion areas with most frequent overlay, among those thresholded at t-value > 3 and that have cluster sizes greater than 10 voxels.

**Table 2:** Cortical, subcortical and white matter projections lesion areas with most frequent overlay in the patient cohort. The regions of interests presented were obtained by overlaying each of three atlases in the combined thresholded lesion map from all the subjects (threshold at T-value > 3 and cluster sizes > 10 voxels).

**JHU white matter atlas**	**Volume (mm^3^)**	**Lesion Overlay (%)**	**Harvard Oxford Cortical atlas**	**Volume (mm^3^)**	**Lesion Overlay (%)**	**Harvard Oxford Subcortical atlas**	**Volume (mm^3^)**	**Lesion Overlay (%)**
Superior corona radiata R	2608	31.8	Lateral Occipital Cortex, superior division R	2840	34.2	Right Cerebral White Matter	13,256	62.2
Posterior limb of internal capsule R	1416	17.3	Angular Gyrus R	1848	22.3	Right Cerebral Cortex	7224	33.9
Superior longitudinal fasciculus R	1264	15.4	Insular Cortex R	1296	15.6	Putamen R	464	2.2
Posterior thalamic radiation (include optic radiation) R	1032	12.6	Middle Temporal Gyrus, temporooccipital part R	1136	13.7	Caudate R	208	0.9
External capsule R	1016	12.4	Lateral Occipital Cortex, inferior division R	592	7.1	Thalamus R	136	0.6
Anterior limb of internal capsule R	336	4.1	Supramarginal Gyrus, posterior division R	240	2.9			
Superior fronto-occipital fasciculus R	176	2.1	Middle Temporal Gyrus, posterior division R	192	2.3			
Retrolenticular part of internal capsule R	128	1.6						
Anterior corona radiata R	128	1.6						

R, right; Note: The total of the lesion overlay percentage might not be exactly 100 because of the ROIs with less than 10 voxels not presented in the table.

### 3.5 Seed-based functional connectivity analyses

#### 3.5.1 Low frequency bands (LF)

**Fig. 3:**
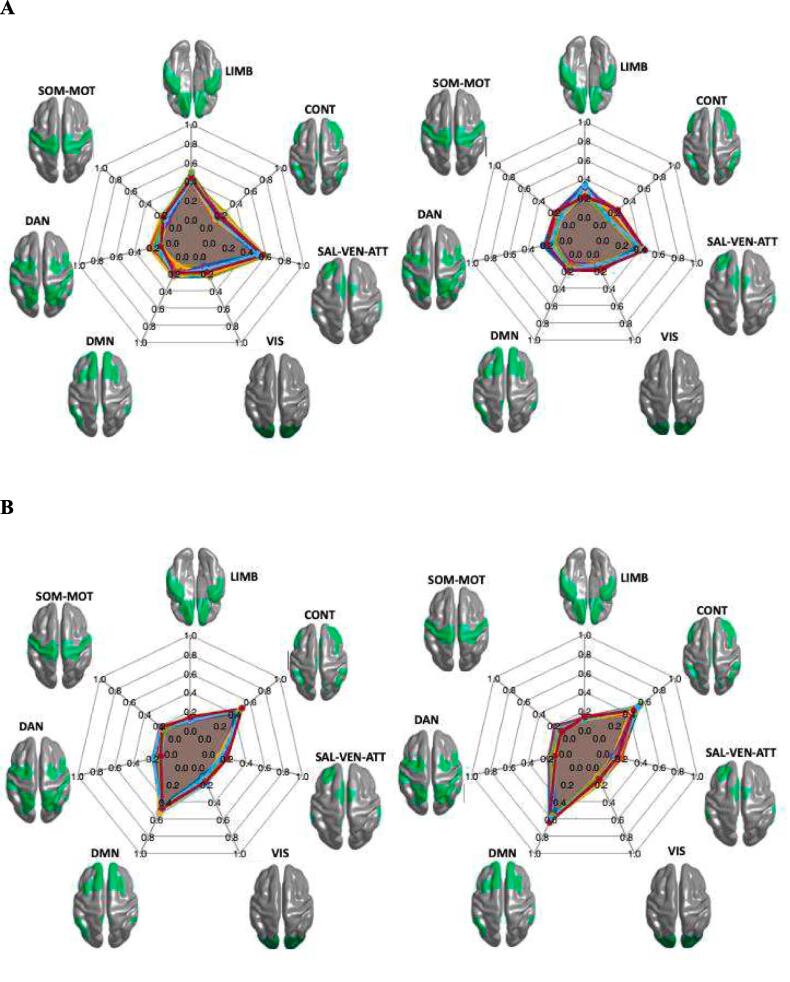
**Directional connectivity spider plots based on LF group.** The connectivity values are shown as a spider plot on the left side for the direction from the identified cortical lesion pattern to the seven networks and on the right side the direction from the seven networks to the cortical lesion pattern. **A.** The group of patients with abnormal LF z-values < -1. **B.** The group of patients with LF z-values > -1.

**Fig. 4:**
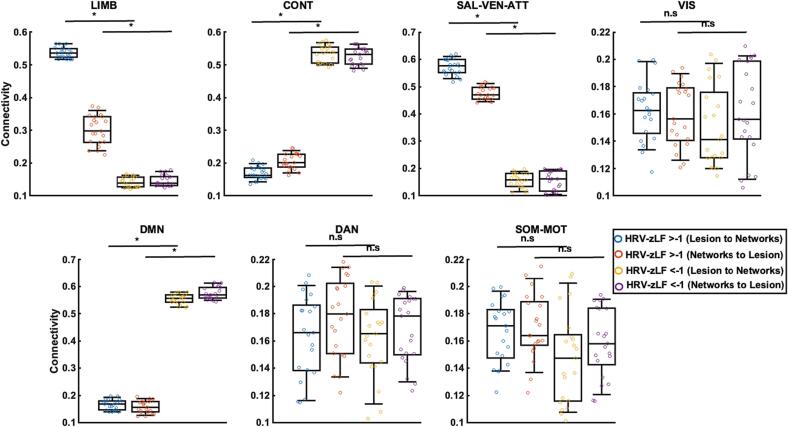
**Connectivity values distribution based on LF group shown separately.** The connectivity values regarding LF are shown separated by network in the seven sub-plots. Each circle represents a patient, and the four colours define the groups in the plots. The statistical difference between the two groups for each direction are indicated by bold lines; * indicates p < 0.001, no significant difference is marked with (n.s).

For the group with abnormal LF-scores (z-score < -1), the brain regions, which correspond to the ICA lesion pattern in these patients, have an influence on the limbic (LIMB, 0.55 ± 0.08) and salience ventral attention networks (SAL-VENT-ATTN; 0.61 ± 0.10) which was stronger than on the other five networks. This influence was reconfirmed by calculating the backward connections from these networks to the lesion pattern (Fig. 3A). In the group without abnormal changes in LF (z-score < -1), the strongest influences could be shown from the ICA lesion pattern to the control network (CONT; 0.54 ± 0.12) and the default mode network (DMN; 0.56 ± 0.09), which again was reconfirmed by backward connection calculation (Fig. 3B). The connectivity between brain regions corresponding to the ICA lesion patters and the resting state networks (both forward and backward connections) was different between the groups with and without pathological LF in LIMB, CONT, SAL-VENT-ATTN and DMN (p < 0.001, Fig. 4).

Additionally, using SVM, the prediction of connectivity values for the group with abnormal LF changes reached significant accuracy (more than 75 %). The testing and overall accuracy was above 80 % for the 10-fold cross validation (Supplementary Fig. 3).

#### 3.5.2 Variation coefficient (VC)

**Fig. 5:**
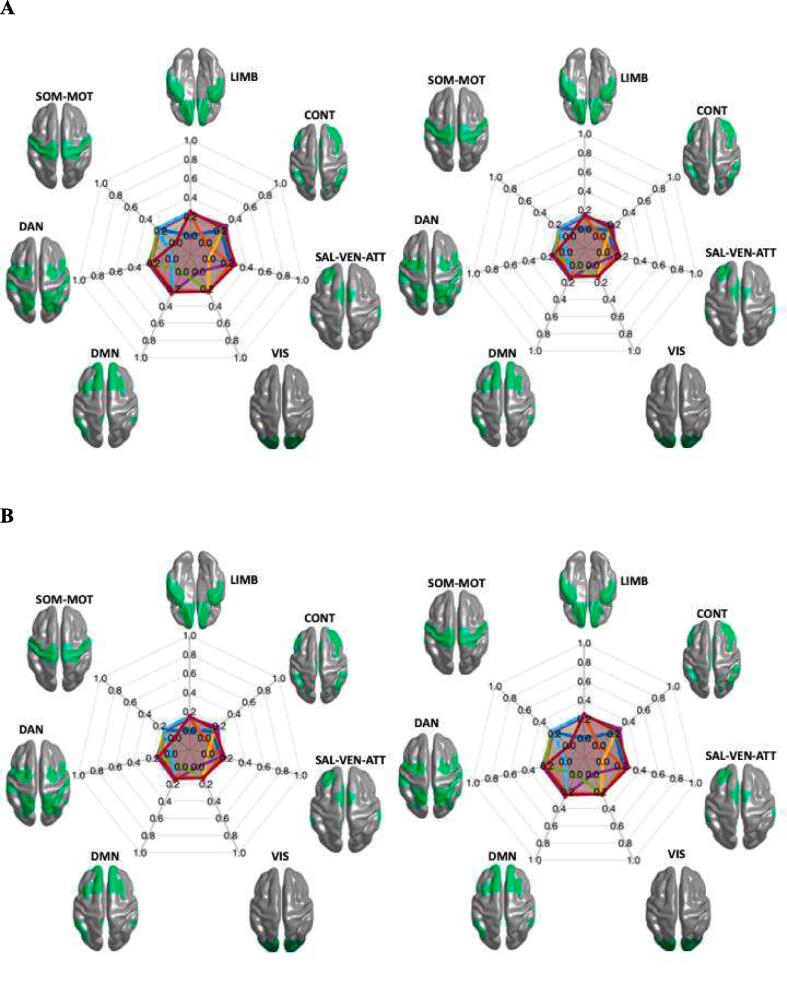
**Directional connectivity spider plots based on VC group**. The connectivity values are shown as a spider plot on the left side for the direction from the identified cortical lesion pattern to the seven networks and on the right side the direction from the seven networks to the cortical lesion pattern. **A.** The group of patients with abnormal VC z-values < -1. **B.** The group of patients with VC z-values > -1.

**Fig. 6:**
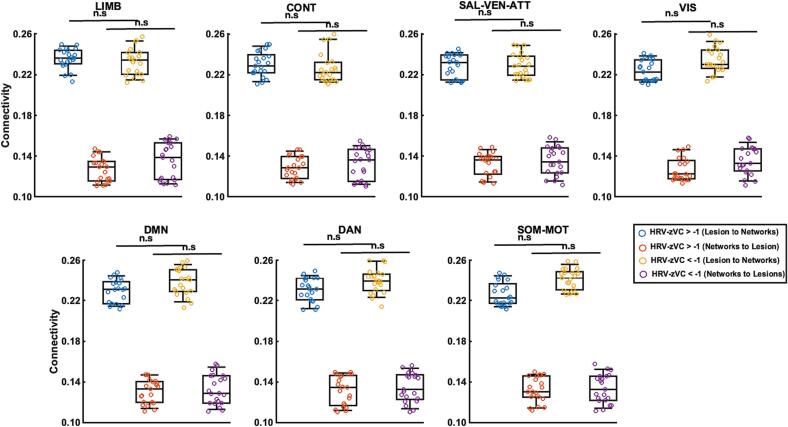
**Connectivity values distribution based on VC group shown separately.** The connectivity values regarding VC are shown separated by the network in the seven sub-plots. Each circle represents a patient, and the four colours defines the groups in the plots. The statistical difference between the two groups for each direction are indicated by bold lines; no significant difference is marked with (n.s).

No different impacts of the ICA lesion pattern could be determined between the groups with and without pathological VC scores (0.08 ± 0.09, p > 0.05, Fig. 5A-B). Throughout, the association of these regions with the seven different networks and from the networks back to the lesions was weak (Fig. 6).

## 4 Discussion

Acute brain lesions might cause significant disturbances of cardiac autonomic function that eventually lead to increased mortality and poor functional outcome (Lees et al., 2018). Given the robustness of the autonomic control of heart rate, which is necessary for survival, and the multitude of brain areas that are related to this autonomic control (Ruffle et al., Oct, 2021), the mechanism explaining how single focal brain lesions could lead to HRV alterations remained obscure. We addressed this question and found that acute brain lesions, which are related to LF power, in particular in the right hemisphere might have effects on resting state cortical networks (Yeo et al., Sep, 2011). This could one mechanism to explain how focal brain lesions influence global bodily function.

### 4.1 HRV after stroke

The present results indicate abnormally reduced cardiac autonomic regulation after acute stroke, with the most significant reductions in the LF (frequency domain) and the VC (time domain), quantified by HRV at rest. The results suggest that acute stroke affects both the sympathetic tone but also vasovagal activity. Although there are studies showing reduced HRV during different provocation tests like deep breathing or standing up (Xiong et al., Dec, 2013) we opted to test HRV at rest because sensorimotor deficits acutely after stroke could interfere with the performance of such tests introducing selection bias and sources of variability. The present investigation is unique in that we controlled for antihypertensive medication including beta blockers.

In LF, which represents both sympathetic and parasympathetic influences on heart rate variability, more patients showed abnormally low values than in VC, which mainly represents parasympathetic influences. Previous results indicate that strokes of similar size to those included in the present study regularly affect function parameters representing both sympathetic and parasympathetic factors but less robustly impact parameters indicating influence of only one branch of the ANS (Xiong et al., 2014). Therefore, similar but also different results were reported before (Chen et al., Mar 2013, Zawadka-Kunikowska et al., Aug 2018). Decreased LF and HF bands in stroke were shown in a small sample of mainly medullary strokes (Meglic et al., 2001). Another study reported unchanged LF bands in acute middle cerebral artery stroke but found changes in the very low frequency band (VLF) (Giubilei et al., 1998). Changes in the VLF were reported to be risk factors for post-stroke infections for unknown reasons (Bramer et al., Dec, 2019). The VLF (range < 0.04 Hz) is influenced by sympathetic and parasympathetic branches similar to the LF (Usui and Nishida, 2017, Baharav et al., Jun 1995).

### 4.2 Lesions and pathological HRV

Stroke lesions associated with LF and VC from all patients formed the same lesion pattern, mainly in the right hemisphere. Although there are reports about a right hemispheric dominance in cardiovascular control (Tokgozoglu et al., Jul 1999, Brunetti et al., Jun 2019) and older studies proposed a right hemispheric lateralization of autonomic control (Meyer et al., 2004), this claim has been challenged by more recent data (Jaremek et al., 2019, Sposato et al., 2020). Honestly, our results could be affected by the low number of patients suffering left hemispheric stroke in our sample due to sampling bias (exclusion of aphasic patients).

That both LF and VC were associated with lesions in mainly the same brain regions could have been expected because of a significant correlation between LF and VC and because in healthy subjects an analysis of gray or white matter co-varying with both HRV parameters revealed grossly the same regions. Only network-based statistics separated parasympathetic from sympathetic HRV parameters (Ruffle et al., Oct, 2021).

Brain regions associated with variance of LF or VC in the present study comprised the temporo-parieto-occipital region, basal ganglia and thalamus, and their interconnections, which include the corona radiata, the (posterior limb of the) internal capsule and the superior longitudinal fasciculus. Previous investigations described different and similar localizations of stroke being associated with reduced HRV spanning from lesions in the frontal lobe, the middle cerebral artery territory (Tokgozoglu et al., Jul, 1999) including basal ganglia (Raphaely-Beer et al., 2020) to the occipital cortex (Wang et al., Sep, 2022).

### 4.3 Functional connectivity to seven networks

Our data so far indicates that there is not a circumscriptive cortex area or white matter tract that controls the autonomic outflow from the brain hemispheres. It rather supports the assumption that HRV must be controlled by networks, which could be affected by different stroke locations. The dysregulation of these networks may cause known cardiovascular complications after stroke, i.e. myocardial injury, myocardial infarction, heart failure, arrhythmias and sudden cardiac death (Sposato et al., 2020). This is why we analyzed the connection of the resulting ICA lesion pattern on seven predefined functional brain functional networks, which were assessed in age-matched healthy subjects. Matching for age is most important because functional brain networks change with age even if subjects are clinically healthy (Damoiseaux, 2017).

We chose to use these seven functional networks (Schaefer et al., 2018) for three main reasons. First, the most well-known and robust rs-fMRI parcellation, which uses global similarity approach, clusters similar functional connectivity patterns regardless of spatial proximity, resulting in parcels with homogeneous (similar) rs-fMRI signals (Schaefer et al., 2018). Second, the atlas is based on 1489 participants and is in concordance with other atlas areas that were defined using histology and visuotopic fMRI. Third, to investigate the global network influence of the stroke lesions patterns, the parcellations were clustered into seven networks using a similar procedure to (Yeo et al., Sep, 2011). In addition, there have been previous studies using this parcellation especially to look at stroke lesion affected networks in the brain (Idesis et al., 2022, Allegra et al., 2021).

We found differences of the impact of the ICA lesion patterns on the 7 networks when contrasting patients with reduced LF versus those with normal LF. We did not find any difference when contrasting patients with reduced and normal VC. In patients with reduced LF, the interaction between lesions and both the LIMB and the SAL-VENT-ATTN was strongest, while in patients with LF in the normal range interactions with the DMN and CONT were predominating. We should not lay too much emphasis on interpreting the clinical consequences of these findings but the differences between both groups are so obvious that the findings support our hypothesis that it is not the lesion but the impact of a lesion on preexisting networks which might cause autonomic disturbances after acute brain damage.

## 5 Limitations

We acknowledge that our study has limitations: 1) The right hemispheric dominance of the lesions in our sample could be affected by sampling bias. Acute left-sided strokes often suffer from aphasia precluding participation in scientific studies. 2) We do not have an explanation for reduced VC in our patients other than there is no direct relation to brain function. 3) We performed a short HRV recording at rest, mainly for clinical reasoning, since patients were recruited from our intensive stroke unit and could be mobilized for limited time. 4) The controls for HRV and rsMRI were different. Although it would be advantageous to derive both measures from the same subjects and thereby to reduce variability, we regard our approach with highly significant interaction despite separate datasets as an argument for the robustness of the findings. 5) Several whole brain functional connectivity atlases exist, e.g. by Shen and colleagues (2013) from 79 healthy normal volunteers (Shen et al., 2013) or by Glaser and colleagues (2016) based on the human connectome project curated with 210 young healthy subjects (Glasser et al., 2016), the selection of which could alter the analysis due to different resolution of the atlases. We used the Schaefer and colleagues (2018) atlas due to three reasons: Firstly, they used a gradient-weighted Markov Random Field (gwMRF) model integrating local gradient and global similarity approaches (Schaefer et al., 2018). Secondly, the atlas was derived using task-fMRI and rs-fMRI across diverse acquisition protocols, and they validated the gwMRF parcellations to be more homogeneous than four previously published parcellations. Thirdly, they were also able to validate them with the boundaries of certain cortical areas defined using histology and visuotopic fMRI. Finally, our findings may be correlative rather than causative for HRV alteration.

## 6 Conclusion

In conclusion, our results indicate that an interaction of brain lesions with existing resting state brain networks predicts the impairment of HRV after stroke, rather than the brain lesion itself.

## 7 Disclosure statement

Dr. Welte-Jzyk reports no disclosures. Dr. Dimova reports no disclosures. Dr. Kronfeld reports no disclosures. Dr. Korczynski reports no disclosures. Dr. Koirala reports no disclosures. Dr. Steenken reports no disclosures. Dr. Kollmann reports no disclosures. Dr. Tüscher reports no disclosures. Dr. Brockman has received speakers’ fees and consulting honoraria from Stryker, Germany, all unrelated to the present work. Dr. Birklein has received speakers’ fees and consulting honoraria from Pfizer, Germany, and Alnylam, Europe, all unrelated to the present work. Dr. Muthuraman reports no disclosures.

## Funding

The work was supported by the Deutsche Forschungsgemeinschaft (DFG, grant numbers Bi579/11–1 to FB, BA4097/3–1 to BB and MU4354/1–1 to MM). The funders had no role in method design, data selection and analysis, decision to publish, or preparation of the manuscript.

## CRediT authorship contribution statement

**Violeta Dimova:** Writing – review & editing, Writing – original draft, Visualization, Methodology, Formal analysis, Data curation, Conceptualization. **Claudia Welte-Jzyk:** Writing – original draft, Formal analysis, Data curation. **Andrea Kronfeld:** Writing – review & editing, Visualization, Formal analysis, Data curation. **Oliver Korczynski:** Writing – review & editing, Visualization, Methodology, Formal analysis. **Bernhard Baier:** Writing – original draft, Visualization, Funding acquisition, Formal analysis, Conceptualization. **Nabin Koirala:** Writing – review & editing, Visualization, Methodology, Formal analysis. **Livia Steenken:** Writing – review & editing, Formal analysis, Data curation. **Bianca Kollmann:** Writing – review & editing, Methodology, Data curation. **Oliver Tüscher:** Writing – review & editing, Visualization, Methodology, Formal analysis. **Marc A. Brockmann:** Writing – review & editing, Visualization, Data curation. **Frank Birklein:** Writing – review & editing, Writing – original draft, Funding acquisition, Formal analysis, Data curation, Conceptualization. **Muthuraman Muthuraman:** .

## Declaration of competing interest

The authors declare that they have no known competing financial interests or personal relationships that could have appeared to influence the work reported in this paper.

## Data Availability

Data will be made available on request.
