# Supplementary material for: Brain connectivity networks underlying resting heart rate variability in acute ischemic stroke
Source: Neuroimage Clin. 2023 Dec 19;41:103558. doi: 10.1016/j.nicl.2023.103558 (PMC10788522; doi:10.1016/j.nicl.2023.103558)
Supplement: Supplementary data 1 [file mmc1.docx]

**Supplementary figure 1**


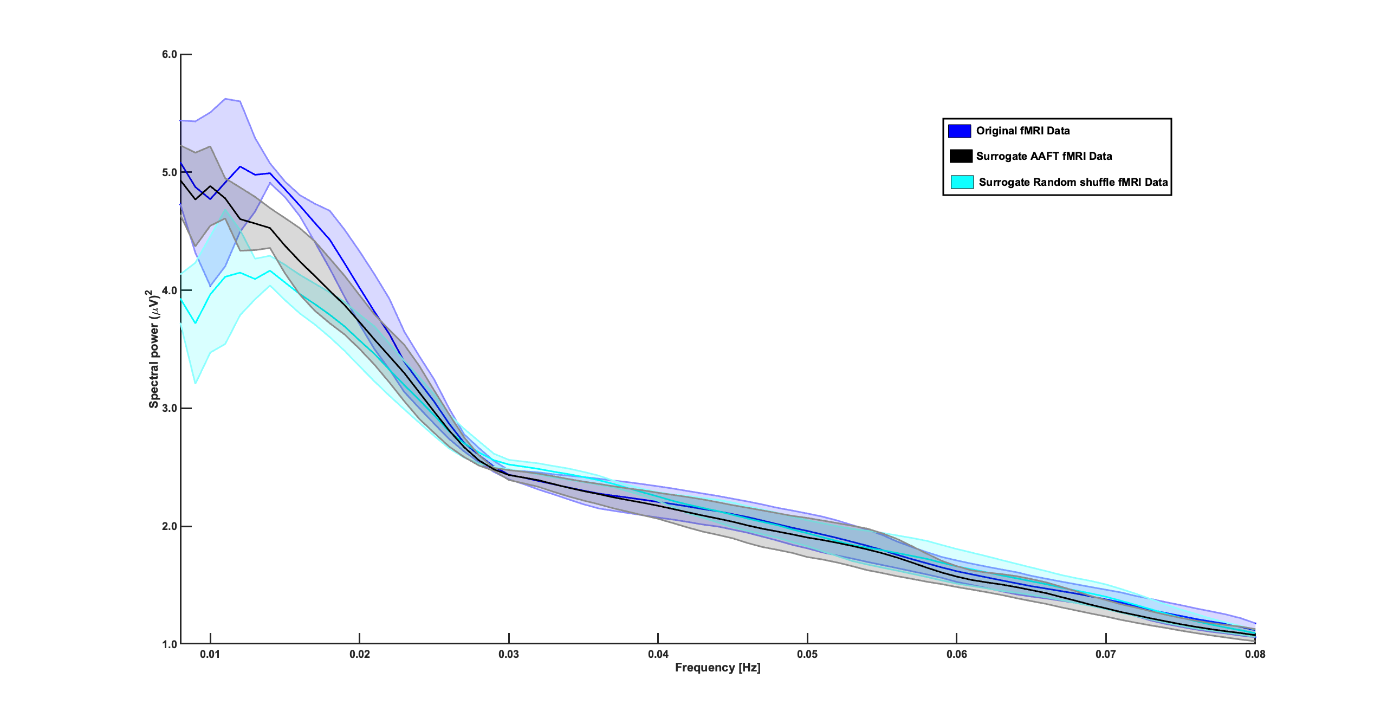


**Supplementary figure 2**


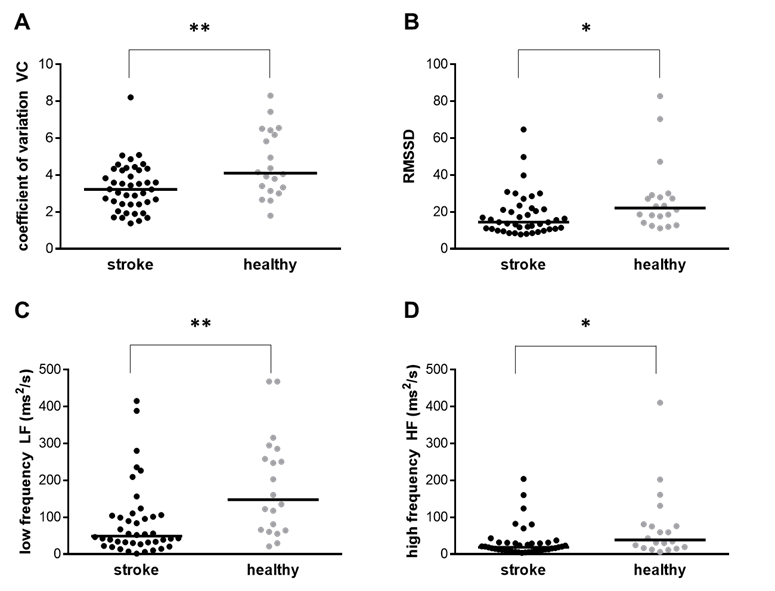


**Supplementary figure 3**


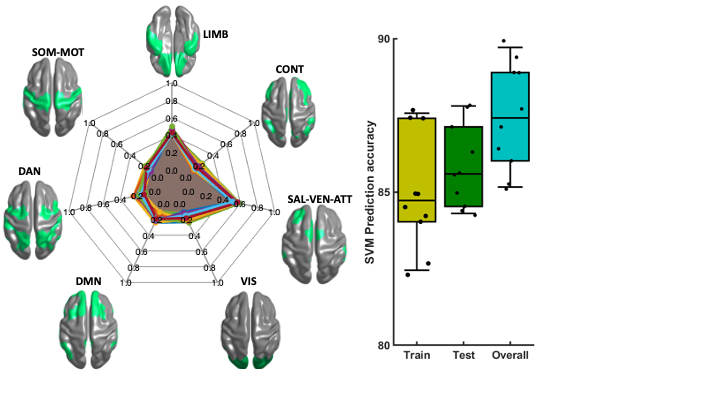


**Supplementary table 1** Influence of antihypertensive medication on HRV parameters. The comparisons among stroke patients and healthy subjects are shown.

|  | Group | | | | Post-hoc comparisons | | | | | |
| --- | --- | --- | --- | --- | --- | --- | --- | --- | --- | --- |
| HRV parameters | Healthy | Stroke | | | Stroke vs healthy | | | Between stroke groups | | |
|  |  | Ø medication | + ß-blocker | Ø ß-blocker | Ø medication | + ß blocker | Ø ß-blocker | Ø medication  vs ß-blocker | Ø medication  vs Ø ß-blocker | ß-blocker  vs Ø ß-blocker |
|  | (n=20) | (n=10) | (n=13) | (n=19) |  |  |  |  |  |  |
|  | mean ± SD | | | | p value | | | | | |
| VC | 4.62 ± 1.80 | 3.23 ± 0.79 | 3.33 ± 1.73 | 3.35 ± 1.27 | **0.020** | **0.019** | **0.011** | 0.874 | 0.832 | 0.965 |
| RMSSD | 27.26 ± 18.97 | 14.90 ± 8.36 | 23.43 ± 16.35 | 16.64 ± 8.39 | **0.030** | 0.457 | **0.024** | 0.163 | 0.758 | 0.193 |
| LF | 185.3 ± 135.28 | 56.92 ± 36.02 | 105.75 ± 111.62 | 90.68 ± 108.20 | **0.004** | **0.050** | **0.010** | 0.302 | 0.441 | 0.708 |
| HF | 74.98 ± 95.14 | 22.16 ± 18.60 | 49 ± 62.91 | 26.31 ± 30 | **0.038** | 0.260 | **0.021** | 0.324 | 0.869 | 0.330 |

VC, coefficient of variation; RMSSD, root mean square of successive differences; LF, low frequency power component; HF, high frequency power component; Ø medication, stroke patients without hypertensive medication; + ß-blocker, stroke patients with hypertensive medication including beta blocker; Ø ß-blocker, stroke patients with other hypertensive medication; post-hoc comparisons based on LSD. Significant p values are highlighted in bold.
